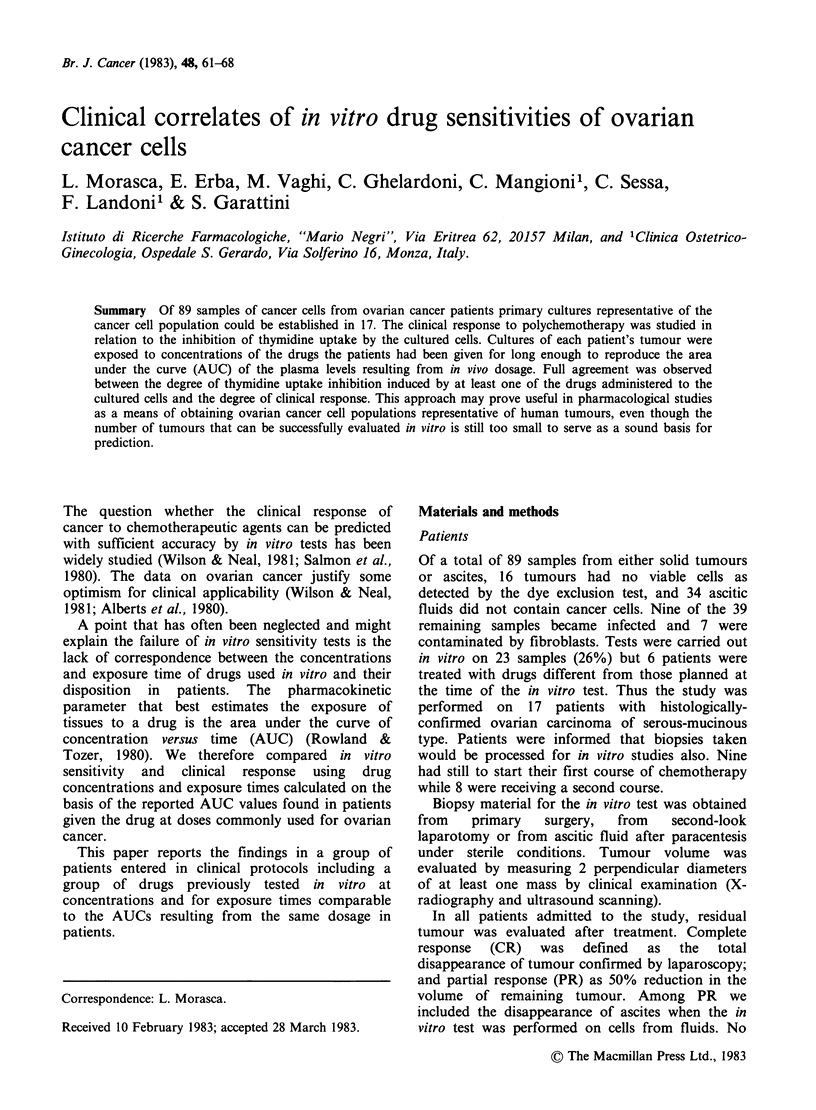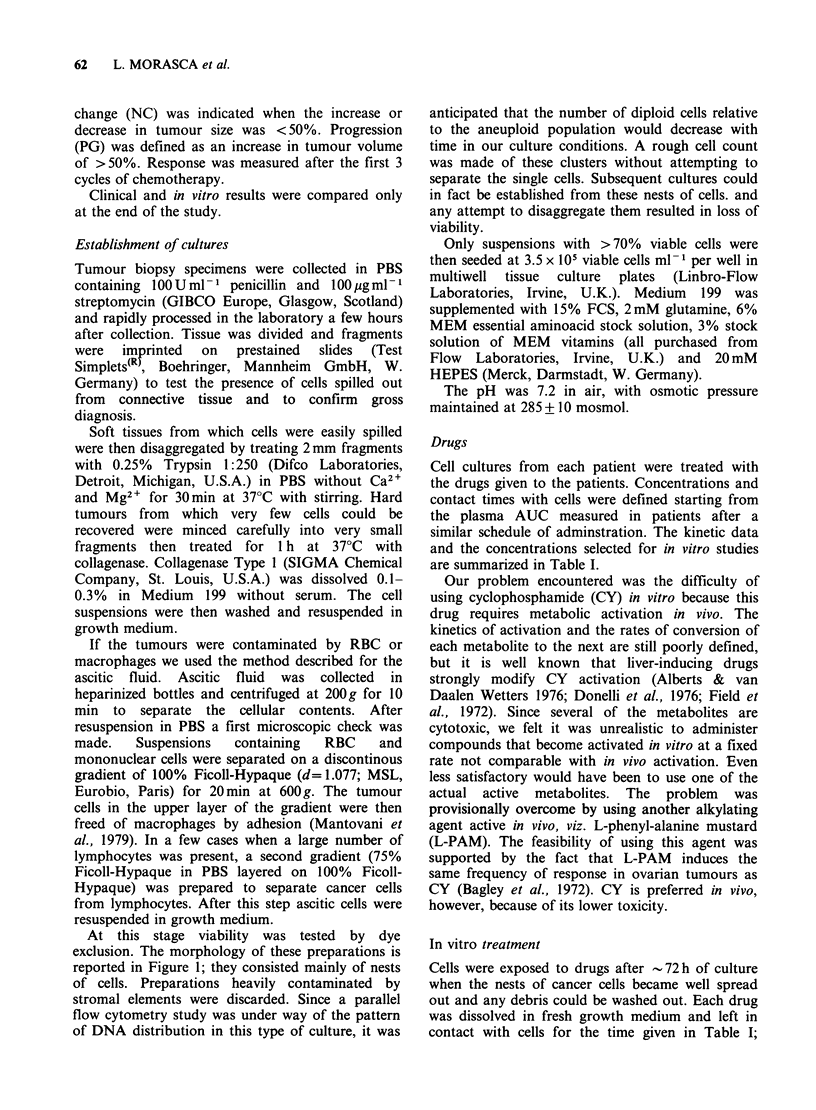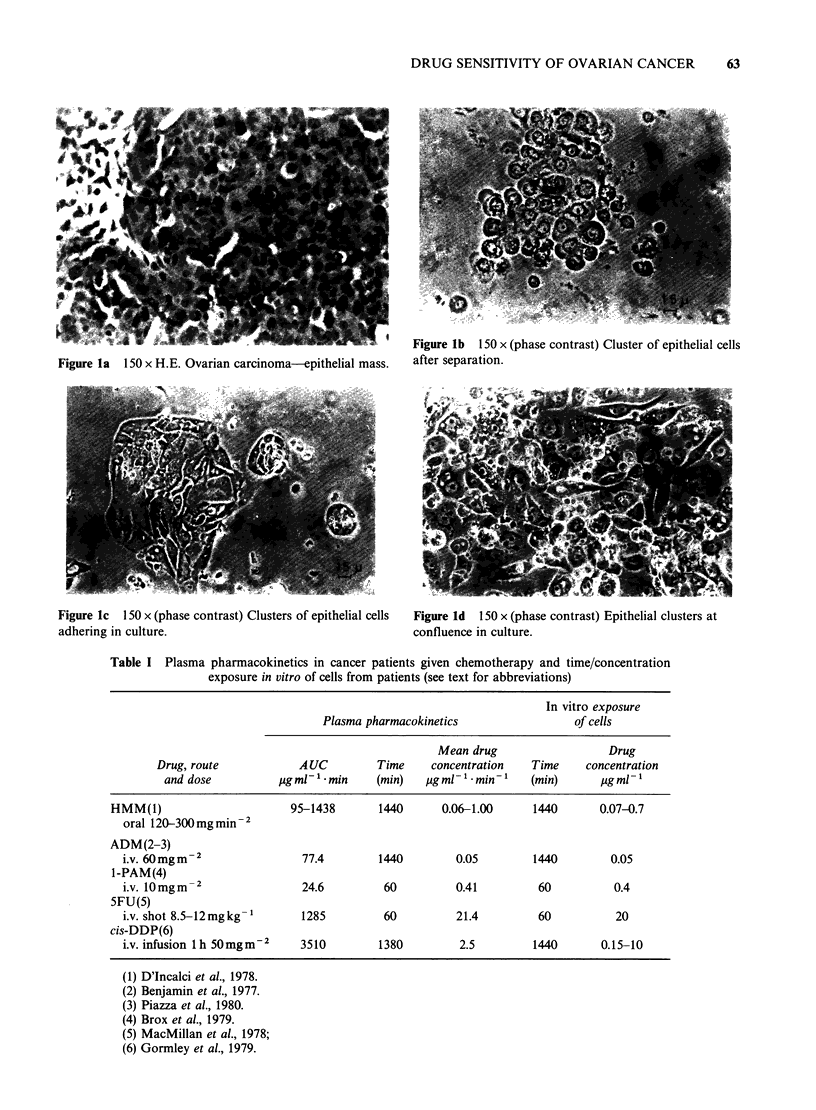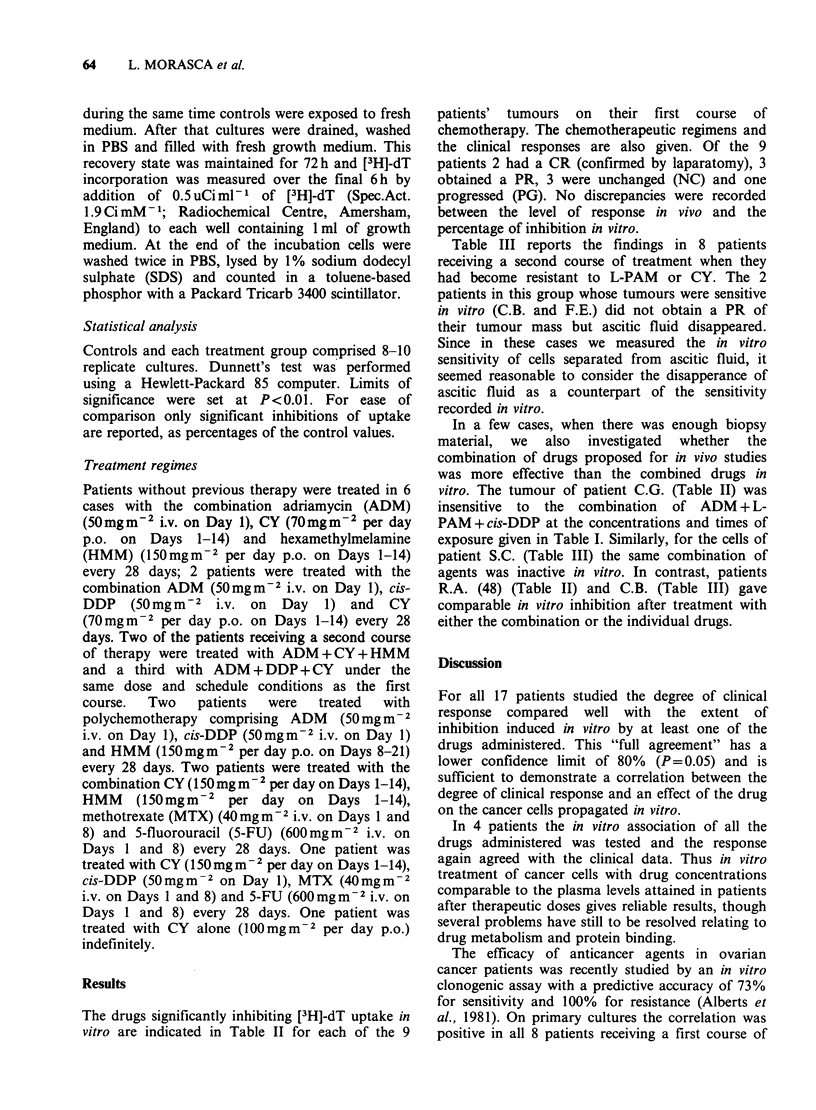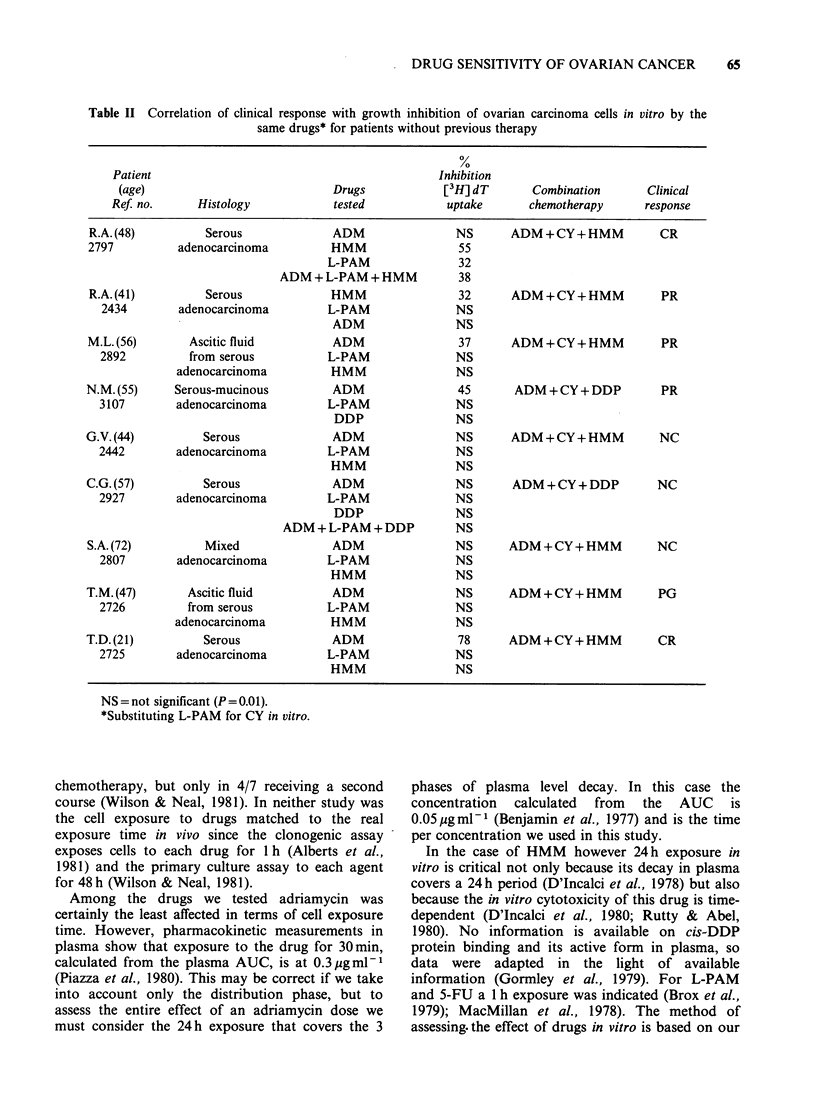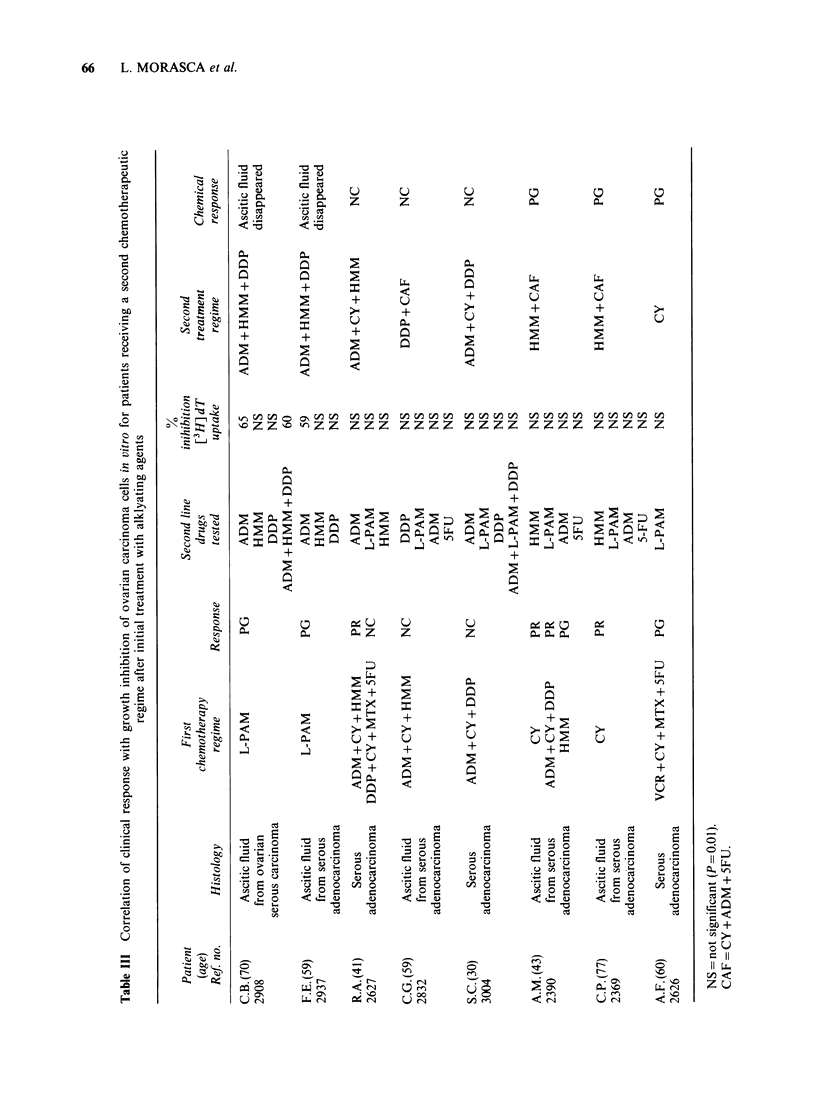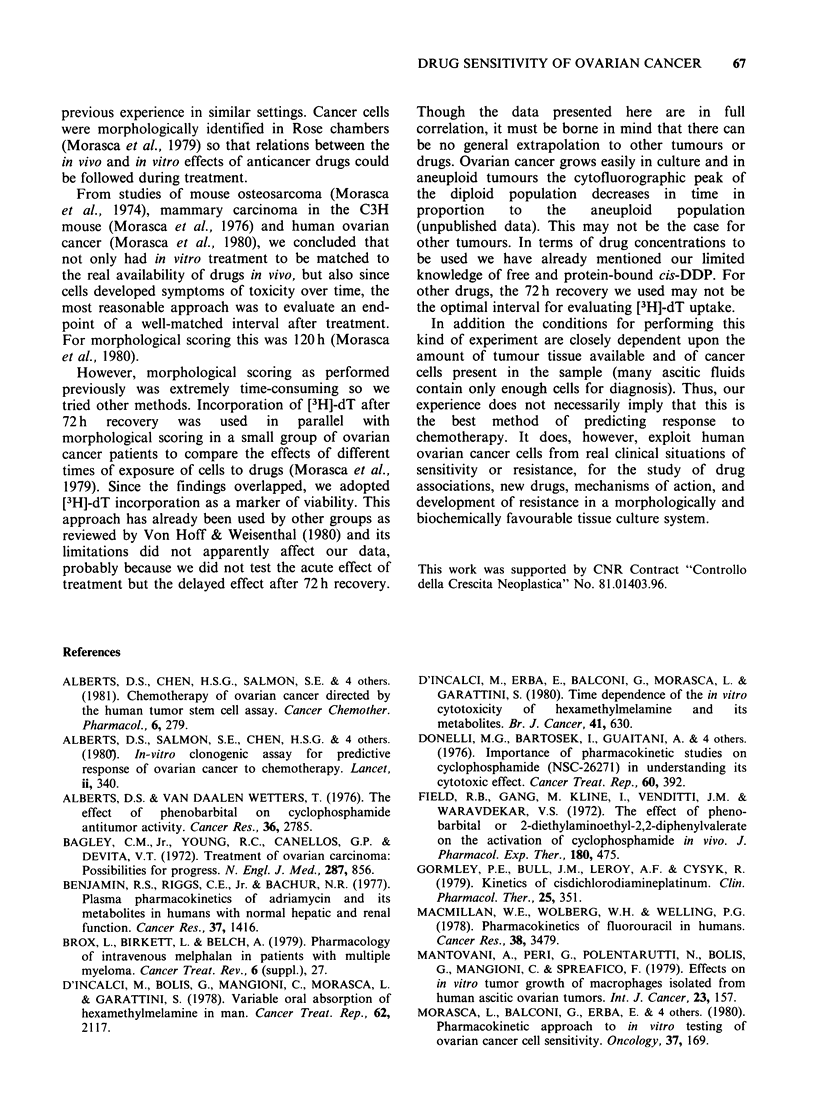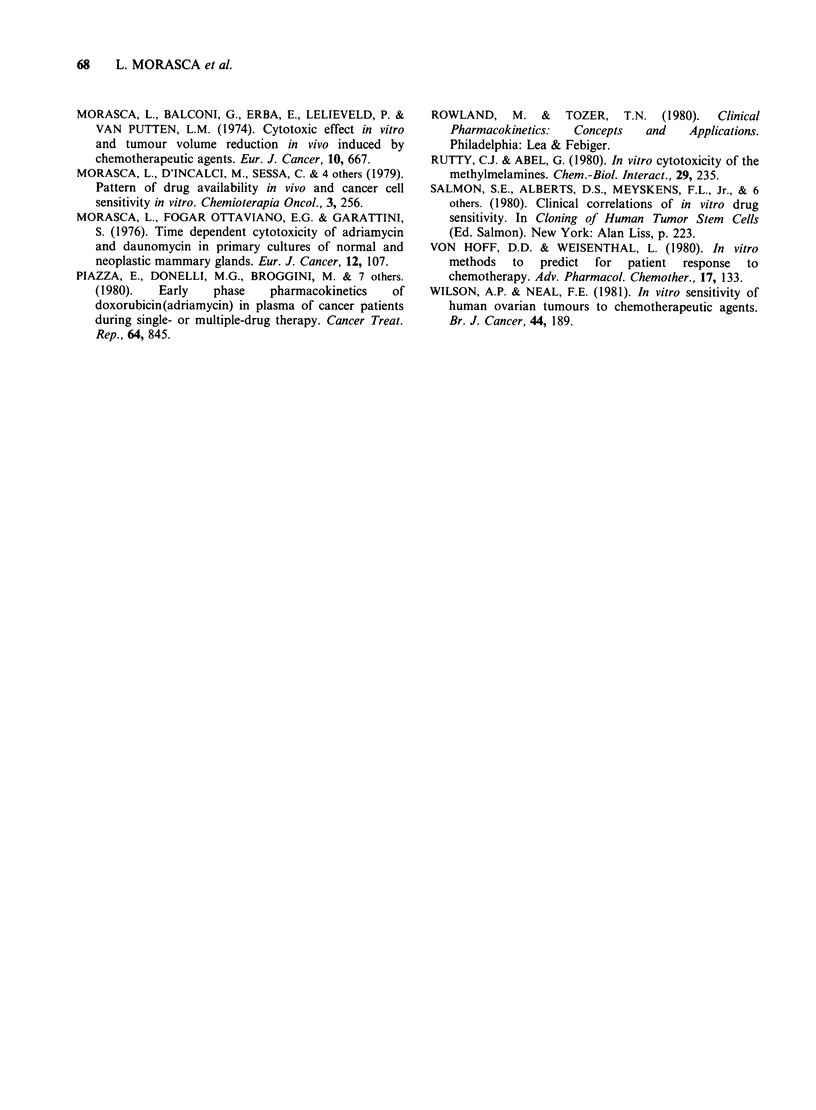# Clinical correlates of in vitro drug sensitivities of ovarian cancer cells.

**DOI:** 10.1038/bjc.1983.157

**Published:** 1983-07

**Authors:** L. Morasca, E. Erba, M. Vaghi, C. Ghelardoni, C. Mangioni, C. Sessa, F. Landoni, S. Garattini

## Abstract

**Images:**


*Br. J. Cancer* (1983), **48**, 61–68

### Clinical correlates of *in vitro* drug sensitivities of ovarian cancer cells

L. Morasca, E. Erba, M. Vaghi, C. Ghelardoni, C. Mangioni[1], C. Sessa, F. Landoni[1] & S. Garattini

*Istituto di Ricerche Farmacologiche, "Mario Negri", Via Eritrea 62, 20157 Milan, and [1]Clinica Ostetrico-Ginecologia, Ospedale S. Gerardo, Via Solferino 16, Monza, Italy.*

**Summary** Of 89 samples of cancer cells from ovarian cancer patients primary cultures representative of the cancer cell population could be established in 17. The clinical response to polychemotherapy was studied in relation to the inhibition of thymidine uptake by the cultured cells. Cultures of each patient's tumour were exposed to concentrations of the drugs the patients had been given for long enough to reproduce the area under the curve (AUC) of the plasma levels resulting from *in vivo* dosage. Full agreement was observed between the degree of thymidine uptake inhibition induced by at least one of the drugs administered to the cultured cells and the degree of clinical response. This approach may prove useful in pharmacological studies as a means of obtaining ovarian cancer cell populations representative of human tumours, even though the number of tumours that can be successfully evaluated *in vitro* is still too small to serve as a sound basis for prediction.

The question whether the clinical response of cancer to chemotherapeutic agents can be predicted with sufficient accuracy by *in vitro* tests has been widely studied (Wilson & Neal, 1981; Salmon *et al.*, 1980). The data on ovarian cancer justify some optimism for clinical applicability (Wilson & Neal, 1981; Alberts *et al.*, 1980).

A point that has often been neglected and might explain the failure of *in vitro* sensitivity tests is the lack of correspondence between the concentrations and exposure time of drugs used *in vitro* and their disposition in patients. The pharmacokinetic parameter that best estimates the exposure of tissues to a drug is the area under the curve of concentration *versus* time (AUC) (Rowland & Tozer, 1980). We therefore compared *in vitro* sensitivity and clinical response using drug concentrations and exposure times calculated on the basis of the reported AUC values found in patients given the drug at doses commonly used for ovarian cancer.

This paper reports the findings in a group of patients entered in clinical protocols including a group of drugs previously tested *in vitro* at concentrations and for exposure times comparable to the AUCs resulting from the same dosage in patients.

Correspondence: L. Morasca.

Received 10 February 1983; accepted 28 March 1983.

### Materials and methods

### *Patients*

Of a total of 89 samples from either solid tumours or ascites, 16 tumours had no viable cells as detected by the dye exclusion test, and 34 ascitic fluids did not contain cancer cells. Nine of the 39 remaining samples became infected and 7 were contaminated by fibroblasts. Tests were carried out *in vitro* on 23 samples (26%) but 6 patients were treated with drugs different from those planned at the time of the *in vitro* test. Thus the study was performed on 17 patients with histologically-confirmed ovarian carcinoma of serous-mucinous type. Patients were informed that biopsies taken would be processed for *in vitro* studies also. Nine had still to start their first course of chemotherapy while 8 were receiving a second course.

Biopsy material for the *in vitro* test was obtained from primary surgery, from second-look laparotomy or from ascitic fluid after paracentesis under sterile conditions. Tumour volume was evaluated by measuring 2 perpendicular diameters of at least one mass by clinical examination (X-radiography and ultrasound scanning).

In all patients admitted to the study, residual tumour was evaluated after treatment. Complete response (CR) was defined as the total disappearance of tumour confirmed by laparoscopy; and partial response (PR) as 50% reduction in the volume of remaining tumour. Among PR we included the disappearance of ascites when the *in vitro* test was performed on cells from fluids. No

© The Macmillan Press Ltd., 1983

change (NC) was indicated when the increase or decrease in tumour size was $<50\%$. Progression (PG) was defined as an increase in tumour volume of $>50\%$. Response was measured after the first 3 cycles of chemotherapy.

Clinical and *in vitro* results were compared only at the end of the study.

### *Establishment of cultures*

Tumour biopsy specimens were collected in PBS containing $100\,U\,ml^{-1}$ penicillin and $100\,\mu g\,ml^{-1}$ streptomycin (GIBCO Europe, Glasgow, Scotland) and rapidly processed in the laboratory a few hours after collection. Tissue was divided and fragments were imprinted on prestained slides (Test Simplets(R), Boehringer, Mannheim GmbH, W. Germany) to test the presence of cells spilled out from connective tissue and to confirm gross diagnosis.

Soft tissues from which cells were easily spilled were then disaggregated by treating 2 mm fragments with 0.25% Trypsin 1:250 (Difco Laboratories, Detroit, Michigan, U.S.A.) in PBS without $Ca^{2+}$ and $Mg^{2+}$ for 30 min at 37°C with stirring. Hard tumours from which very few cells could be recovered were minced carefully into very small fragments then treated for 1 h at 37°C with collagenase. Collagenase Type 1 (SIGMA Chemical Company, St. Louis, U.S.A.) was dissolved 0.1–0.3% in Medium 199 without serum. The cell suspensions were then washed and resuspended in growth medium.

If the tumours were contaminated by RBC or macrophages we used the method described for the ascitic fluid. Ascitic fluid was collected in heparinized bottles and centrifuged at 200 *g* for 10 min to separate the cellular contents. After resuspension in PBS a first microscopic check was made. Suspensions containing RBC and mononuclear cells were separated on a discontinous gradient of 100% Ficoll-Hypaque ($d = 1.077$; MSL, Eurobio, Paris) for 20 min at 600 *g*. The tumour cells in the upper layer of the gradient were then freed of macrophages by adhesion (Mantovani *et al.*, 1979). In a few cases when a large number of lymphocytes was present, a second gradient (75% Ficoll-Hypaque in PBS layered on 100% Ficoll-Hypaque) was prepared to separate cancer cells from lymphocytes. After this step ascitic cells were resuspended in growth medium.

At this stage viability was tested by dye exclusion. The morphology of these preparations is reported in Figure 1; they consisted mainly of nests of cells. Preparations heavily contaminated by stromal elements were discarded. Since a parallel flow cytometry study was under way of the pattern of DNA distribution in this type of culture, it was anticipated that the number of diploid cells relative to the aneuploid population would decrease with time in our culture conditions. A rough cell count was made of these clusters without attempting to separate the single cells. Subsequent cultures could in fact be established from these nests of cells. and any attempt to disaggregate them resulted in loss of viability.

Only suspensions with $>70\%$ viable cells were then seeded at $3.5 \times 10^5$ viable cells $ml^{-1}$ per well in multiwell tissue culture plates (Linbro-Flow Laboratories, Irvine, U.K.). Medium 199 was supplemented with 15% FCS, 2 mM glutamine, 6% MEM essential aminoacid stock solution, 3% stock solution of MEM vitamins (all purchased from Flow Laboratories, Irvine, U.K.) and 20 mM HEPES (Merck, Darmstadt, W. Germany).

The pH was 7.2 in air, with osmotic pressure maintained at $285 \pm 10$ mosmol.

### *Drugs*

Cell cultures from each patient were treated with the drugs given to the patients. Concentrations and contact times with cells were defined starting from the plasma AUC measured in patients after a similar schedule of adminstration. The kinetic data and the concentrations selected for *in vitro* studies are summarized in Table I.

Our problem encountered was the difficulty of using cyclophosphamide (CY) *in vitro* because this drug requires metabolic activation *in vivo*. The kinetics of activation and the rates of conversion of each metabolite to the next are still poorly defined, but it is well known that liver-inducing drugs strongly modify CY activation (Alberts & van Daalen Wetters 1976; Donelli *et al.*, 1976; Field *et al.*, 1972). Since several of the metabolites are cytotoxic, we felt it was unrealistic to administer compounds that become activated *in vitro* at a fixed rate not comparable with *in vivo* activation. Even less satisfactory would have been to use one of the actual active metabolites. The problem was provisionally overcome by using another alkylating agent active *in vivo*, *viz.* L-phenyl-alanine mustard (L-PAM). The feasibility of using this agent was supported by the fact that L-PAM induces the same frequency of response in ovarian tumours as CY (Bagley *et al.*, 1972). CY is preferred *in vivo*, however, because of its lower toxicity.

### In vitro *treatment*

Cells were exposed to drugs after ~72 h of culture when the nests of cancer cells became well spread out and any debris could be washed out. Each drug was dissolved in fresh growth medium and left in contact with cells for the time given in Table I;

**Figure 1a** 150 × H.E. Ovarian carcinoma—epithelial mass.

**Figure 1b** 150 × (phase contrast) Cluster of epithelial cells after separation.

**Figure 1c** 150 × (phase contrast) Clusters of epithelial cells adhering in culture.

**Figure 1d** 150 × (phase contrast) Epithelial clusters at confluence in culture.

**Table I** Plasma pharmacokinetics in cancer patients given chemotherapy and time/concentration exposure *in vitro* of cells from patients (see text for abbreviations)

| | *Plasma pharmacokinetics* | | | In vitro *exposure of cells* | |
|---|---|---|---|---|---|
| *Drug, route and dose* | *AUC* $\mu g\,ml^{-1}\cdot min$ | *Time (min)* | *Mean drug concentration* $\mu g\,ml^{-1}\cdot min^{-1}$ | *Time (min)* | *Drug concentration* $\mu g\,ml^{-1}$ |
| HMM(1) oral 120–300 mg min$^{-2}$ | 95–1438 | 1440 | 0.06–1.00 | 1440 | 0.07–0.7 |
| ADM(2–3) | | | | | |
| i.v. 60 mg m$^{-2}$ | 77.4 | 1440 | 0.05 | 1440 | 0.05 |
| 1-PAM(4) | | | | | |
| i.v. 10 mg m$^{-2}$ | 24.6 | 60 | 0.41 | 60 | 0.4 |
| 5FU(5) | | | | | |
| i.v. shot 8.5–12 mg kg$^{-1}$ | 1285 | 60 | 21.4 | 60 | 20 |
| *cis*-DDP(6) | | | | | |
| i.v. infusion 1 h 50 mg m$^{-2}$ | 3510 | 1380 | 2.5 | 1440 | 0.15–10 |

(1) D'Incalci *et al.*, 1978.
(2) Benjamin *et al.*, 1977.
(3) Piazza *et al.*, 1980.
(4) Brox *et al.*, 1979.
(5) MacMillan *et al.*, 1978;
(6) Gormley *et al.*, 1979.

during the same time controls were exposed to fresh medium. After that cultures were drained, washed in PBS and filled with fresh growth medium. This recovery state was maintained for 72 h and [$^3$H]-dT incorporation was measured over the final 6 h by addition of 0.5 uCi ml$^{-1}$ of [$^3$H]-dT (Spec.Act. 1.9 Ci mM$^{-1}$; Radiochemical Centre, Amersham, England) to each well containing 1 ml of growth medium. At the end of the incubation cells were washed twice in PBS, lysed by 1% sodium dodecyl sulphate (SDS) and counted in a toluene-based phosphor with a Packard Tricarb 3400 scintillator.

*Statistical analysis*

Controls and each treatment group comprised 8–10 replicate cultures. Dunnett's test was performed using a Hewlett-Packard 85 computer. Limits of significance were set at $P<0.01$. For ease of comparison only significant inhibitions of uptake are reported, as percentages of the control values.

*Treatment regimes*

Patients without previous therapy were treated in 6 cases with the combination adriamycin (ADM) (50 mg m$^{-2}$ i.v. on Day 1), CY (70 mg m$^{-2}$ per day p.o. on Days 1–14) and hexamethylmelamine (HMM) (150 mg m$^{-2}$ per day p.o. on Days 1–14) every 28 days; 2 patients were treated with the combination ADM (50 mg m$^{-2}$ i.v. on Day 1), *cis*-DDP (50 mg m$^{-2}$ i.v. on Day 1) and CY (70 mg m$^{-2}$ per day p.o. on Days 1–14) every 28 days. Two of the patients receiving a second course of therapy were treated with ADM + CY + HMM and a third with ADM + DDP + CY under the same dose and schedule conditions as the first course. Two patients were treated with polychemotherapy comprising ADM (50 mg m$^{-2}$ i.v. on Day 1), *cis*-DDP (50 mg m$^{-2}$ i.v. on Day 1) and HMM (150 mg m$^{-2}$ per day p.o. on Days 8–21) every 28 days. Two patients were treated with the combination CY (150 mg m$^{-2}$ per day on Days 1–14), HMM (150 mg m$^{-2}$ per day on Days 1–14), methotrexate (MTX) (40 mg m$^{-2}$ i.v. on Days 1 and 8) and 5-fluorouracil (5-FU) (600 mg m$^{-2}$ i.v. on Days 1 and 8) every 28 days. One patient was treated with CY (150 mg m$^{-2}$ per day on Days 1–14), *cis*-DDP (50 mg m$^{-2}$ on Day 1), MTX (40 mg m$^{-2}$ i.v. on Days 1 and 8) and 5-FU (600 mg m$^{-2}$ i.v. on Days 1 and 8) every 28 days. One patient was treated with CY alone (100 mg m$^{-2}$ per day p.o.) indefinitely.

### Results

The drugs significantly inhibiting [$^3$H]-dT uptake *in vitro* are indicated in Table II for each of the 9 patients' tumours on their first course of chemotherapy. The chemotherapeutic regimens and the clinical responses are also given. Of the 9 patients 2 had a CR (confirmed by laparatomy), 3 obtained a PR, 3 were unchanged (NC) and one progressed (PG). No discrepancies were recorded between the level of response *in vivo* and the percentage of inhibition *in vitro*.

Table III reports the findings in 8 patients receiving a second course of treatment when they had become resistant to L-PAM or CY. The 2 patients in this group whose tumours were sensitive *in vitro* (C.B. and F.E.) did not obtain a PR of their tumour mass but ascitic fluid disappeared. Since in these cases we measured the *in vitro* sensitivity of cells separated from ascitic fluid, it seemed reasonable to consider the disapperance of ascitic fluid as a counterpart of the sensitivity recorded *in vitro*.

In a few cases, when there was enough biopsy material, we also investigated whether the combination of drugs proposed for *in vivo* studies was more effective than the combined drugs *in vitro*. The tumour of patient C.G. (Table II) was insensitive to the combination of ADM + L-PAM + *cis*-DDP at the concentrations and times of exposure given in Table I. Similarly, for the cells of patient S.C. (Table III) the same combination of agents was inactive *in vitro*. In contrast, patients R.A. (48) (Table II) and C.B. (Table III) gave comparable *in vitro* inhibition after treatment with either the combination or the individual drugs.

### Discussion

For all 17 patients studied the degree of clinical response compared well with the extent of inhibition induced *in vitro* by at least one of the drugs administered. This "full agreement" has a lower confidence limit of 80% ($P=0.05$) and is sufficient to demonstrate a correlation between the degree of clinical response and an effect of the drug on the cancer cells propagated *in vitro*.

In 4 patients the *in vitro* association of all the drugs administered was tested and the response again agreed with the clinical data. Thus *in vitro* treatment of cancer cells with drug concentrations comparable to the plasma levels attained in patients after therapeutic doses gives reliable results, though several problems have still to be resolved relating to drug metabolism and protein binding.

The efficacy of anticancer agents in ovarian cancer patients was recently studied by an *in vitro* clonogenic assay with a predictive accuracy of 73% for sensitivity and 100% for resistance (Alberts *et al.*, 1981). On primary cultures the correlation was positive in all 8 patients receiving a first course of

**Table II** Correlation of clinical response with growth inhibition of ovarian carcinoma cells *in vitro* by the same drugs* for patients without previous therapy

| *Patient (age) Ref. no.* | *Histology* | *Drugs tested* | *% Inhibition [$^3H$]dT uptake* | *Combination chemotherapy* | *Clinical response* |
|---|---|---|---|---|---|
| R.A. (48) | Serous | ADM | NS | ADM+CY+HMM | CR |
| 2797 | adenocarcinoma | HMM | 55 | | |
| | | L-PAM | 32 | | |
| | | ADM+L-PAM+HMM | 38 | | |
| R.A. (41) | Serous | HMM | 32 | ADM+CY+HMM | PR |
| 2434 | adenocarcinoma | L-PAM | NS | | |
| | | ADM | NS | | |
| M.L. (56) | Ascitic fluid | ADM | 37 | ADM+CY+HMM | PR |
| 2892 | from serous | L-PAM | NS | | |
| | adenocarcinoma | HMM | NS | | |
| N.M. (55) | Serous-mucinous | ADM | 45 | ADM+CY+DDP | PR |
| 3107 | adenocarcinoma | L-PAM | NS | | |
| | | DDP | NS | | |
| G.V. (44) | Serous | ADM | NS | ADM+CY+HMM | NC |
| 2442 | adenocarcinoma | L-PAM | NS | | |
| | | HMM | NS | | |
| C.G. (57) | Serous | ADM | NS | ADM+CY+DDP | NC |
| 2927 | adenocarcinoma | L-PAM | NS | | |
| | | DDP | NS | | |
| | | ADM+L-PAM+DDP | NS | | |
| S.A. (72) | Mixed | ADM | NS | ADM+CY+HMM | NC |
| 2807 | adenocarcinoma | L-PAM | NS | | |
| | | HMM | NS | | |
| T.M. (47) | Ascitic fluid | ADM | NS | ADM+CY+HMM | PG |
| 2726 | from serous | L-PAM | NS | | |
| | adenocarcinoma | HMM | NS | | |
| T.D. (21) | Serous | ADM | 78 | ADM+CY+HMM | CR |
| 2725 | adenocarcinoma | L-PAM | NS | | |
| | | HMM | NS | | |

NS = not significant ($P = 0.01$).
*Substituting L-PAM for CY *in vitro*.

chemotherapy, but only in 4/7 receiving a second course (Wilson & Neal, 1981). In neither study was the cell exposure to drugs matched to the real exposure time *in vivo* since the clonogenic assay exposes cells to each drug for 1 h (Alberts *et al.*, 1981) and the primary culture assay to each agent for 48 h (Wilson & Neal, 1981).

Among the drugs we tested adriamycin was certainly the least affected in terms of cell exposure time. However, pharmacokinetic measurements in plasma show that exposure to the drug for 30 min, calculated from the plasma AUC, is at 0.3 $\mu$g ml$^{-1}$ (Piazza *et al.*, 1980). This may be correct if we take into account only the distribution phase, but to assess the entire effect of an adriamycin dose we must consider the 24 h exposure that covers the 3 phases of plasma level decay. In this case the concentration calculated from the AUC is 0.05 $\mu$g ml$^{-1}$ (Benjamin *et al.*, 1977) and is the time per concentration we used in this study.

In the case of HMM however 24 h exposure *in vitro* is critical not only because its decay in plasma covers a 24 h period (D'Incalci *et al.*, 1978) but also because the *in vitro* cytotoxicity of this drug is time-dependent (D'Incalci *et al.*, 1980; Rutty & Abel, 1980). No information is available on *cis*-DDP protein binding and its active form in plasma, so data were adapted in the light of available information (Gormley *et al.*, 1979). For L-PAM and 5-FU a 1 h exposure was indicated (Brox *et al.*, 1979); MacMillan *et al.*, 1978). The method of assessing the effect of drugs *in vitro* is based on our

**Table III** Correlation of clinical response with growth inhibition of ovarian carcinoma cells *in vitro* for patients receiving a second chemotherapeutic regime after initial treatment with alklyating agents

| *Patient (age) Ref. no.* | *Histology* | *First chemotherapy regime* | *Response* | *Second line drugs tested* | *% inihibition [$^3H$] dT uptake* | *Second treatment regime* | *Chemical response* |
|---|---|---|---|---|---|---|---|
| C.B. (70) 2908 | Ascitic fluid from ovarian serous carcinoma | L-PAM | PG | ADM | 65 | ADM + HMM + DDP | Ascitic fluid disappeared |
| | | | | HMM | NS | | |
| | | | | DDP | NS | | |
| | | | | ADM + HMM + DDP | 60 | | |
| F.E. (59) 2937 | Ascitic fluid from serous adenocarcinoma | L-PAM | PG | ADM | 59 | ADM + HMM + DDP | Ascitic fluid disappeared |
| | | | | HMM | NS | | |
| | | | | DDP | NS | | |
| R.A. (41) 2627 | Serous adenocarcinoma | ADM + CY + HMM | PR | ADM | NS | ADM + CY + HMM | NC |
| | | DDP + CY + MTX + 5FU | NC | L-PAM | NS | | |
| | | | | HMM | NS | | |
| C.G. (59) 2832 | Ascitic fluid from serous adenocarcinoma | ADM + CY + HMM | NC | DDP | NS | DDP + CAF | NC |
| | | | | L-PAM | NS | | |
| | | | | ADM | NS | | |
| | | | | 5FU | NS | | |
| S.C. (30) 3004 | Serous adenocarcinoma | ADM + CY + DDP | NC | ADM | NS | ADM + CY + DDP | NC |
| | | | | L-PAM | NS | | |
| | | | | DDP | NS | | |
| | | | | ADM + L-PAM + DDP | NS | | |
| A.M. (43) 2390 | Ascitic fluid from serous adenocarcinoma | CY | PR | HMM | NS | HMM + CAF | PG |
| | | ADM + CY + DDP | PR | L-PAM | NS | | |
| | | HMM | PG | ADM | NS | | |
| | | | | 5FU | NS | | |
| C.P. (77) 2369 | Ascitic fluid from serous adenocarcinoma | CY | PR | HMM | NS | HMM + CAF | PG |
| | | | | L-PAM | NS | | |
| | | | | ADM | NS | | |
| | | | | 5-FU | NS | | |
| A.F. (60) 2626 | Serous adenocarcinoma | VCR + CY + MTX + 5FU | PG | L-PAM | NS | CY | PG |

NS = not significant ($P = 0.01$).

CAF = CY + ADM + 5FU.

previous experience in similar settings. Cancer cells were morphologically identified in Rose chambers (Morasca *et al.*, 1979) so that relations between the *in vivo* and *in vitro* effects of anticancer drugs could be followed during treatment.

From studies of mouse osteosarcoma (Morasca *et al.*, 1974), mammary carcinoma in the C3H mouse (Morasca *et al.*, 1976) and human ovarian cancer (Morasca *et al.*, 1980), we concluded that not only had *in vitro* treatment to be matched to the real availability of drugs *in vivo*, but also since cells developed symptoms of toxicity over time, the most reasonable approach was to evaluate an end-point of a well-matched interval after treatment. For morphological scoring this was 120 h (Morasca *et al.*, 1980).

However, morphological scoring as performed previously was extremely time-consuming so we tried other methods. Incorporation of [$^3$H]-dT after 72 h recovery was used in parallel with morphological scoring in a small group of ovarian cancer patients to compare the effects of different times of exposure of cells to drugs (Morasca *et al.*, 1979). Since the findings overlapped, we adopted [$^3$H]-dT incorporation as a marker of viability. This approach has already been used by other groups as reviewed by Von Hoff & Weisenthal (1980) and its limitations did not apparently affect our data, probably because we did not test the acute effect of treatment but the delayed effect after 72 h recovery.

Though the data presented here are in full correlation, it must be borne in mind that there can be no general extrapolation to other tumours or drugs. Ovarian cancer grows easily in culture and in aneuploid tumours the cytofluorographic peak of the diploid population decreases in time in proportion to the aneuploid population (unpublished data). This may not be the case for other tumours. In terms of drug concentrations to be used we have already mentioned our limited knowledge of free and protein-bound *cis*-DDP. For other drugs, the 72 h recovery we used may not be the optimal interval for evaluating [$^3$H]-dT uptake.

In addition the conditions for performing this kind of experiment are closely dependent upon the amount of tumour tissue available and of cancer cells present in the sample (many ascitic fluids contain only enough cells for diagnosis). Thus, our experience does not necessarily imply that this is the best method of predicting response to chemotherapy. It does, however, exploit human ovarian cancer cells from real clinical situations of sensitivity or resistance, for the study of drug associations, new drugs, mechanisms of action, and development of resistance in a morphologically and biochemically favourable tissue culture system.

This work was supported by CNR Contract "Controllo della Crescita Neoplastica" No. 81.01403.96.

### References

ALBERTS, D.S., CHEN, H.S.G., SALMON, S.E. & 4 others. (1981). Chemotherapy of ovarian cancer directed by the human tumor stem cell assay. *Cancer Chemother. Pharmacol.*, **6**, 279.

ALBERTS, D.S., SALMON, S.E., CHEN, H.S.G. & 4 others. (1980). *In-vitro* clonogenic assay for predictive response of ovarian cancer to chemotherapy. *Lancet*, **ii**, 340.

ALBERTS, D.S. & VAN DAALEN WETTERS, T. (1976). The effect of phenobarbital on cyclophosphamide antitumor activity. *Cancer Res.*, **36**, 2785.

BAGLEY, C.M., Jr., YOUNG, R.C., CANELLOS, G.P. & DEVITA, V.T. (1972). Treatment of ovarian carcinoma: Possibilities for progress. *N. Engl. J. Med.*, **287**, 856.

BENJAMIN, R.S., RIGGS, C.E., Jr. & BACHUR, N.R. (1977). Plasma pharmacokinetics of adriamycin and its metabolites in humans with normal hepatic and renal function. *Cancer Res.*, **37**, 1416.

BROX, L., BIRKETT, L. & BELCH, A. (1979). Pharmacology of intravenous melphalan in patients with multiple myeloma. *Cancer Treat. Rev.*, **6** (suppl.), 27.

D'INCALCI, M., BOLIS, G., MANGIONI, C., MORASCA, L. & GARATTINI, S. (1978). Variable oral absorption of hexamethylmelamine in man. *Cancer Treat. Rep.*, **62**, 2117.

D'INCALCI, M., ERBA, E., BALCONI, G., MORASCA, L. & GARATTINI, S. (1980). Time dependence of the *in vitro* cytotoxicity of hexamethylmelamine and its metabolites. *Br. J. Cancer*, **41**, 630.

DONELLI, M.G., BARTOSEK, I., GUAITANI, A. & 4 others. (1976). Importance of pharmacokinetic studies on cyclophosphamide (NSC-26271) in understanding its cytotoxic effect. *Cancer Treat. Rep.*, **60**, 392.

FIELD, R.B., GANG, M. KLINE, I., VENDITTI, J.M. & WARAVDEKAR, V.S. (1972). The effect of phenobarbital or 2-diethylaminoethyl-2,2-diphenylvalerate on the activation of cyclophosphamide *in vivo*. *J. Pharmacol. Exp. Ther.*, **180**, 475.

GORMLEY, P.E., BULL, J.M., LEROY, A.F. & CYSYK, R. (1979). Kinetics of cisdichlorodiamineplatinum. *Clin. Pharmacol. Ther.*, **25**, 351.

MACMILLAN, W.E., WOLBERG, W.H. & WELLING, P.G. (1978). Pharmacokinetics of fluorouracil in humans. *Cancer Res.*, **38**, 3479.

MANTOVANI, A., PERI, G., POLENTARUTTI, N., BOLIS, G., MANGIONI, C. & SPREAFICO, F. (1979). Effects on *in vitro* tumor growth of macrophages isolated from human ascitic ovarian tumors. *Int. J. Cancer*, **23**, 157.

MORASCA, L., BALCONI, G., ERBA, E. & 4 others. (1980). Pharmacokinetic approach to *in vitro* testing of ovarian cancer cell sensitivity. *Oncology*, **37**, 169.

MORASCA, L., BALCONI, G., ERBA, E., LELIEVELD, P. & VAN PUTTEN, L.M. (1974). Cytotoxic effect *in vitro* and tumour volume reduction *in vivo* induced by chemotherapeutic agents. *Eur. J. Cancer,* **10,** 667.

MORASCA, L., D'INCALCI, M., SESSA, C. & 4 others (1979). Pattern of drug availability *in vivo* and cancer cell sensitivity *in vitro. Chemioterapia Oncol.,* **3,** 256.

MORASCA, L., FOGAR OTTAVIANO, E.G. & GARATTINI, S. (1976). Time dependent cytotoxicity of adriamycin and daunomycin in primary cultures of normal and neoplastic mammary glands. *Eur. J. Cancer,* **12,** 107.

PIAZZA, E., DONELLI, M.G., BROGGINI, M. & 7 others. (1980). Early phase pharmacokinetics of doxorubicin(adriamycin) in plasma of cancer patients during single- or multiple-drug therapy. *Cancer Treat. Rep.,* **64,** 845.

ROWLAND, M. & TOZER, T.N. (1980). *Clinical Pharmacokinetics: Concepts and Applications.* Philadelphia: Lea & Febiger.

RUTTY, C.J. & ABEL, G. (1980). *In vitro* cytotoxicity of the methylmelamines. *Chem.-Biol. Interact.,* **29,** 235.

SALMON, S.E., ALBERTS, D.S., MEYSKENS, F.L., Jr., & 6 others. (1980). Clinical correlations of *in vitro* drug sensitivity. In *Cloning of Human Tumor Stem Cells* (Ed. Salmon). New York: Alan Liss, p. 223.

VON HOFF, D.D. & WEISENTHAL, L. (1980). *In vitro* methods to predict for patient response to chemotherapy. *Adv. Pharmacol. Chemother.,* **17,** 133.

WILSON, A.P. & NEAL, F.E. (1981). *In vitro* sensitivity of human ovarian tumours to chemotherapeutic agents. *Br. J. Cancer,* **44,** 189.